# Quality Characteristics of Healthy Dry Fermented Sausages Formulated with a Mixture of Olive and Chia Oil Structured in Oleogel or Emulsion Gel as Animal Fat Replacer

**DOI:** 10.3390/foods9060830

**Published:** 2020-06-24

**Authors:** Tatiana Pintado, Susana Cofrades

**Affiliations:** Institute of Food Science, Technology and Nutrition (ICTAN-CSIC), José Antonio Novais 10, 28040 Madrid, Spain; scofrades@ictan.csic.es

**Keywords:** oleogel, emulsion gel, dry fermented sausages, healthier lipid content, chia oil, olive oil

## Abstract

The present work evaluates the suitability of beeswax oleogels and emulsion gel prepared with a healthy lipid mixture (olive and chia oils) as pork fat replacers for the development of a dry fermented meat product (fuet). Because these systems offer various possibilities, this study has compared their effect on the nutritional quality and sensory acceptability of fuets and their behaviour with regard to technological properties and microbiological and oxidative stability during 30 days of chilled storage. This strategy allowed products with an improved fatty acid profile and a 12-fold decrease of the polyunsaturated fatty acids (PUFA) n-6/n-3 ratio, as compared to the control samples. Irrespective of the structuring method used as animal fat replacer, reformulated samples showed a good oxidative status during chilled storage. In general, no differences that depended on the use of oleogel or emulsion gel were observed in the technological properties and microbiological status, so the choice of one or the other would be conditioned by other factors than the characteristics that the product develops. However, further studies are needed to improve the sensory attributes of the reformulated samples.

## 1. Introduction

Fuet is a type of small-caliber non-acid fermented sausage from northeast Spain made with pork meat, pork fat and various seasonings. Traditionally, fermented sausages were considered safe and healthy foods, but nowadays these products have been associated with health hazards owing to the presence of some components such as saturated fats [1]. In this regard, several options have been assayed to improve lipid content in meat products based on the incorporation of vegetable oils, directly added into the meat matrix, stabilized in oil-in-water emulsion, etc. [2]. However, the interest in alternative technologies has been increasing, and therefore efforts have been made to develop healthy solid fats for foods, attaching importance to their ability to help to promote health and wellbeing [3]. Oleogels and emulsion gels are two different solid oil structured systems that offer interesting characteristics for use as animal fat replacers in the development of healthy meat products [4,5,6,7,8]. In oleogels, liquid oil is transformed into a ‘gel-like’ structure by using an organogelator, while emulsion gels may be generated from a stable liquid-like emulsion by gelling the continuous phase [3]. Regardless of the type of structured oil system, it is desirable to select an oil or a mixture of oils with a healthy fatty acid profile (reduced saturated fats, rich in unsaturated fats and good Σ polyunsaturated fatty acids (PUFA) n-6/ΣPUFA n-3 ratio, etc.), according to recommendations [9,10]. Accordingly, a mixture of olive oil, which is characterized by its high oleic fatty acid [11], and chia oil, which is the richest known botanical source of n-3 linolenic acid and does not contain any of the antinutritional compounds (total linamarin, linustatin and neolinustatin) or vitamin B6 antagonist factors that are present in other commercially-available sources of n-3 linolenic acid [12], could provide a way of obtaining new solid lipid materials with healthy fatty acid as animal fat replacers.

Some studies have been carried out to improve the fatty acid profile of cooked (frankfurter sausages) or fresh meat products (patties, longanizas, merguez) by using solid lipid material based on emulsion gels or oleogels [6,13,14,15,16,17,18,19,20,21,22,23]. However, there are very few studies of this kind on fermented meat products. For example, for this purpose linseed emulsion gel [24,25] or oleogel [26] was used to replace animal fat in dry fermented sausages. But we have found no studies that compare the use of emulsion gels and oleogels as animal fat replacers to improve the lipid content in meat products of any kind.

Accordingly, taking into account the particularity of this type of dry fermented meat product owing to the reactions that occur during the ripening process, the present study aimed to evaluate the quality of a functional fermented meat product (fuet) as a function of the olive-chia oil mixture structured as an oleogel or emulsion gel, used as animal fat replacer. The behaviour during one month of chilled storage was also evaluated.

## 2. Materials and Methods

### 2.1. Oleogel and Emulsion Gel Preparation

Two different animal fat replacers based on solid-structuring oil systems were made: an oleogel (OG) and an emulsion gel (EG). OG consisted mostly of oil (90%), while EG had half that oil content (45%). In both, the lipid phase consisted of a mixture of 80% olive oil (Carbonell Virgen Extra, SOS Cuétara, S.A., Madrid, Spain) and 20% chia oil (Primaria Premium Raw Materials, S.L., Valencia, Spain). The olive oil contained 13% saturated fatty acid (SFA), 75% monounsaturated fatty acid (MUFA) and 8% polyunsaturated fatty acid (PUFA), as reported by Delgado-Pando [27]. According to the information provided by the supplier, the chia oil contained 10% SFA, 5% MUFA and 80% PUFA.

Beeswax (Manuel Riesgo, S.A., Madrid, Spain), which was used as an organogelator in the OG formulation, was prepared as previously described by Gomez-Estaca [6]. Briefly, the oil mixture (90%) and beeswax (10%) were heated (65 °C) under constant stirring (500 rpm) in a food processor (Vorwerk Thermomix TM 31, Wuppertal, Germany) until complete melting and mixing. The resulting solution was then immediately poured into metal containers under pressure to compact it and prevent air bubbles, and it was stored at 3 ± 1 °C after standing for 60 min at room temperature in darkness.

EG was prepared as described by Pintado [7]. Briefly, soy protein isolate (10%) (Manuel Riesgo, S.A., Madrid, Spain) was mixed with water in a Thermomix TM 31 (Wuppertal, Germany) food processor (30 s, approx. 5600 rpm). Then, as a gelling agent, gelatin (3%) (type B, 200–220 bloom) from Manuel Riesgo, S.A. (Madrid, Spain) was added and combined (15 s, approx. 5600 rpm). The final mixture was mixed at approx. 5600 rpm with gradual addition of the appropriate amount (45%) of the oil mixture described previously. Finally, it was placed in metal containers under pressure to compact it and prevent air bubbles, and stored in a chilled room at 3 ± 1 °C for 20 h until use.

### 2.2. Fuet Design and Preparation

Sufficient fresh post-rigor pork meat (a mixture of biceps femoris, semimembranosus, semitendinosus, gracilis and adductor muscles) and pork backfat were obtained from a local market. Both the pork meat and the backfat were vacuum packed in batches of approximately 1000 and 500 g respectively, to be frozen and stored at −20 °C until used (less than one month).

Four different fuet-type dry fermented sausages were formulated (Table 1) in a pilot plant. Two formulations without replacement of pork backfat were prepared as references: one with normal fat content (NF/C) and the other with reduced fat content (RF/C). Additionally, two reduced-fat fuets were formulated, in which pork backfat was partially replaced by oleogel (RF/OG) or emulsion gel (RF/EG). Although the level of fat replacement was the same, different amounts of OG and EG had to be added to obtain a similar lipid content.

Previously thawed pork meat and pork backfat (~18 h at 2 ± 2 °C) and the new lipid materials (OG in RF/OG and EG in RF/EG) were minced to a particle size of 6 mm (Van Dall S.r.l., model FTSIII, Treviglio, Italy). The ingredients for each formulation (Table 1) were placed in a mixer (MAINCA, Barcelona, Spain) and homogenized for 1 min. Half of the water and a commercial seasoning preparation for fuet (COMPLET FUETIB CU-425, Pilarica, Valencia, Spain) were added to the mixture and it was mixed for 1 min. Then the other half of the water and seasoning were added and the result was mixed again for 2 min. The mixture was stuffed (manual stuffer, MAINCA, Barcelona, Spain) into 34/36 mm-diameter natural pork casings (Julio Criado Gómez, S.A., Madrid, Spain), resulting in sausages weighing about 200 g. The sausages were dipped in a meat surface starter suspension of *Penicillium nalgiovense* and *Penicillium candidum* (TEXEL NEO 1 Danisco, DuPont™, Madrid, Spain) prepared according to the manufacturer’s instructions. The sausages were placed in a ripening cabinet (BINDER model KBF 240, Tuttlingen, Germany) under the following conditions: 2 days at 19 °C and 80–85% relative humidity (RH) and 15 days at 13 °C and 75–80% RH. These conditions were set for all the products in order to have no other variables, despite the fact that the water content conditions the ripening process of fermented products [28]. The fuets were packed in plastic bags under aerobic conditions and kept in chilled storage (2 ± 2 °C) for 30 days.

Samples from each formulation were taken at 0 (the end of the ripening process and the beginning of storage), 15 and 30 days of chilled storage for analysis.

### 2.3. Processing Losses

Losses were calculated by weight difference during the fuet ripening period and expressed as a percentage of the initial weight.

### 2.4. Chemical Composition and Energy Value of Fuets

The chemical composition of the fuets was analyzed at the end of the ripening period. Each analysis was performed three times. Moisture and ash content were determined using official methods [29]. A LECO FP-2000 Nitrogen Determinator (Leco Corporation, St Joseph, MI, USA) was used to evaluate protein content and fat level was measured in accordance with Bligh and Dyer [30]. The energy value was calculated on the basis of 9 kcal/g for fat and 4 kcal/g for protein.

The fatty acid content was evaluated in triplicate by saponification and bimethylation according to Lee [31] in samples previously freeze-dried (Lyophilizer Telstar Cryodos Equipment, Tarrasa, Spain). The analysis of fatty acid methyl ester (FAME) was carried out on an Agilent gas chromatograph (Model 7820A, Santa Clara, CA, USA) fitted with a GC-7 Agilent HP-88 capillary column (60 m × 250 µm × 0.2 μm) using a flame ionization detector The temperature of the injector and the detector was 250 and 260 °C respectively. On the other hand, the temperature profile of the oven was 125 °C, increasing by 8 °C/min to 145 °C (held for 26 min) and 2 °C/min to 220 °C (held for 5 min). C13:0 was used as internal patron and for the identification of fatty acids, that was carried out by comparison of the retention times, it was used the standard 47015-U Supelco PUFA No.2 Animal Source (Sigma-Aldrich Co., St. Louis, MO, USA). Fatty acids were expressed as g of fatty acid/100 g product.

### 2.5. Technological Properties

Technological properties were evaluated during the chilled storage of the fuets, at 0, 15 and 30 days.

The pH was determined (in quadruplicate) at room temperature in water in a ratio of 1:10 (w/v) using a 827 Metrohm pH-meter (Metrohm AG, Zofingen, Switzerland).

Water activity (Aw) was measured (in triplicate) at 25 °C, after removing the casing, in a LabMaster-aw instrument (model 1119977, Novasina AG, Lachen SZ, Switzerland).

Colour was measured (ten times) in fuet cross-sections using a Konica Minolta CM-3500 D spectrophotometer (Konica Minolta Business Technologies, Tokyo, Japan) set to D65 illuminant/10° observer. The CIELAB colour space was used to obtain the colour coordinates L* (black (0) to white (100)), a* (green (–) to red (+)), and b* (blue (–) to yellow (+)).

Texture profile analysis (TPA), as described by Bourne [32], was carried out using a TA-XTplus Texture Analyzer (Stable Micro Systems Ltd., Godalming, UK) equipped with a 30 kg load cell. Six cores (diameter = 12 mm, height = 20 mm) per sample were axially compressed to 50% of their original height at a crosshead speed of 0.8 mm/s to calculate hardness (N). The tests were performed on the samples at room temperature immediately after refrigeration at 3 °C.

### 2.6. Lipid Oxidation

The fuets were assessed for oxidative stability by measuring secondary oxidation products, based on changes in concentrations of thiobarbituric acid-reactive substances (TBARs) and the main volatile aldehyde compounds formed by lipid oxidation [33].

TBARs, which were expressed as mg malonaldehyde (MDA)/kg fuet based on a standard curve prepared from 1,1,3,3-tetraethoxypropane in advance, were determined according to Delgado-Pando [34],. Volatile compounds of the fuet samples were extracted by solid phase micro-extraction and determined according to Alejandre [24]. The gas chromatograph (Agilent, model 6890N, Santa Clara, CA, USA) was equipped with a 5973 Mass Selective Detector and it used a DB-WAXetr polyethylene glycol capillary column (60 m × 320 µm × 0.25 μm). For the analysis the oven temperature was set initially at 40 °C (4 min hold), increased to 110 °C at 4 °C/min, to 180 °C at 6 °C/min, and to 240 °C at 8 °C/min (15 min hold). Helium was used as a carrier gas at 1.3 mL/min; injector and detector temperatures were held at 250 and 240 °C, respectively. Identification of the peaks was based on comparison of their mass spectra with the spectra of a commercial library (Wiley 7th edition and NIST/EPA/NIH 02 mass spectral library) and by comparison of their retention times with those of standard compounds. For semi-quantitative purposes, peak area was measured by integration of the total ion current of the spectra. Results were expressed as area/sample weight (g) × 10^3^.

Determinations for each sample, volatile compounds, and TBARs were performed in triplicate at day 0 and after 30 days of chilled storage.

### 2.7. Microbiological Analysis

Total viable counts (TVC) and lactic acid bacteria (LAB) were evaluated as described Pintado et al. [19]. For results exposure, all microbial counts were converted to logarithms of colony-forming units per gram (Log cfu/g).

### 2.8. Sensory Analysis

The sensory analysis was carried out with a panel of 30 assessors selected from the Institute of Food Science, Technology and Nutrition (ICTAN-CSIC) staff. These people were chosen because they are acquainted with meat products and the terminology used for the analysis. For samples preparation, the fuets were cut into 3-mm-thick slices. Two slices per sample were presented to the panellists, who were instructed to rinse their mouth with bread and water between samples. The sensory attributes (general appearance, odour, flavour, texture and overall acceptability) were evaluated on a 10-point scale, 0 being considered as “dislike strongly” and 10 as “like strongly”. The panellists were also asked to make any comments that they considered relevant about their sensory perception of the samples.

### 2.9. Statistical Analysis

The whole experiment was performed twice. Statistical tests were made employing the SPSS computer program (v24 SPSS Statistical Software, Inc., Chicago, IL, USA). One-way and/or two-way analyses of variance (ANOVA) were performed. Differences between pairs of means were assessed on the basis of confidence intervals using Tukey’s Honestly-significant-difference (HSD) test. The level of significance was *p* ≤ 0.05.

## 3. Results and Discussion

### 3.1. Processing Losses

**Figure 1:**
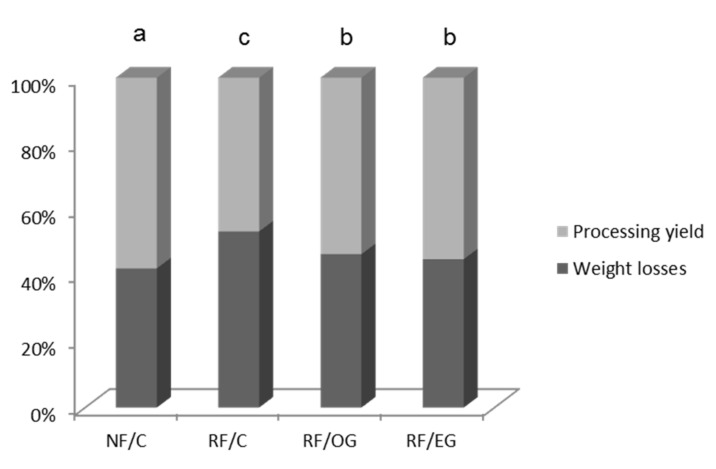
Weight losses and the processing yield of the fuets as a consequence of the ripening process. Normal fat (NF/C) and reduced-fat (RF/C) dry fermented sausages (fuet) formulated with all-animal fat. Reduced-fat fuets reformulated by partially replacing (80%) pork backfat with oleogel (RF/OG) or emulsion gel (RF/EG). Different letters indicate significant differences by formulation in weight losses and processing yield (*p* < 0.05).

At the end of the ripening process, the losses that products suffered were calculated to evaluate the yield for the various products as a consequence of the reformulation strategy (Figure 1). Samples with all-animal fat had the highest weight losses, 53.4% (RF/C), and the lowest, 42.2% (NF/C). Several authors [28,35,36] have observed higher losses in reduced-fat fermented sausages than in sausages with normal fat. In the present study, the strategy of reducing fat and improving the lipid profile by using oleogel (OG) and emulsion gel (EG) led to products with better binding properties than when only the fat content was reduced (RF/C) (Figure 1). No differences were observed between samples with OG or EG despite the higher quantity of water added directly during the preparation of RF/OG than in the case of RF/EG, in which water was stabilized or entrapped in an emulsion (Table 1).

### 3.2. Chemical Composition and Energy Value

The fuet composition (Table 2) was mainly influenced by the formulation (Table 1). However, for this type of meat product, the ripening process should be taken into account because during this period there is a high water loss (Figure 1), which is one of the characteristics that determine the final composition of the product because it results in concentration of the components.

As expected on the basis of the fuet formulations (Table 1), products with two different fat levels were obtained (Table 2). The two strategies used in this work, the replacement of animal fat by water alone or by the use of structured oils, OG (RF/OG) and EG (RF/EG), led to products with similar (*p* > 0.05) fat content (Table 2). Moisture content increased significantly as a result of the reduction in fat level, as other authors have found in dry fermented sausages [24,37]. Despite the differences in water losses (Figure 1), no significant differences were found in moisture content that depended on the type of fat used as the lipid source (all-animal-fat, OG, or EG). Reduced all-animal-fat fuet (RF/C) had the highest (*p* < 0.05) ash content, probably because it had the highest losses (Figure 1) during processing. The protein levels of the fuets were between 31.17% and 37.74% (Table 2). The use of oleogel and emulsion gel as fat replacers resulted in samples with different (*p* < 0.05) protein contents, probably because of the use of soy protein isolate as emulsifier in the preparation of the emulsion gel.

Both strategies, the pork backfat reduction and the partial pork backfat replacement by oleogel and emulsion gel systems, improved the fatty lipid profile, with decreased SFA and increased PUFA (*p* < 0.05). With regard to SFA, the use of OG and EG as animal fat replacers significantly reduced the myristic, palmitic and stearic acid contents in the fuets by more than half compared to the control (NF/C) (Table 2). The highest (*p* < 0.05) MUFA content was in the control samples (NF/C). However, MUFA represented 54% of total fat in NF/C, whereas in RF/OG and RF/EG MUFA content was approximately 60% of total fat. Oleic acid was the main fatty acid in all samples (Table 2), which is consistent with reports for fatty acid composition in pork fat [38] and in olive oil [27], which was the main oil used in the development of OG and EG. The RF/OG and RF/EG products showed the highest (*p* < 0.05) PUFA content, with a notable increase in α-linolenic fatty acid (ALA) in both samples owing to the presence of chia oil, which is the richest known botanical source of n-3 linolenic acid [12]. Consequently, owing to the technological advantages that chia seed and chia flour offer and their high lipid content (30–35%), both products have also been used (added directly or in emulsion or emulsion gel) to improve the fatty acid profile of various meat products, such as frankfurters, burgers, longanizas, etc. [19,39,40,41]. The PUFA/SFA ratio is one of the main parameters currently used to assess the nutritional quality of the lipid fraction of foods, and a PUFA/SFA ratio above 0.4 is recommended [38]. The PUFA/SFA ratio in the all-animal-fat samples (N/FC and R/FC) was around 0.2 (Table 2), which is consistent with reports by other authors concerning conventional meat products [20,42], whereas replacement of pork fat by the new healthy lipid materials (OG and EG) increased this ratio (*p* < 0.05) to 0.6 (Table 2), thus complying with the recommendations. The PUFA n-6/n-3 ratio is also of great interest, because diets with high PUFA n-6/n-3 ratios promote the pathogenesis of many diseases (cardiovascular diseases, cancer, etc.), whereas increased n-3 PUFA content exerts a suppressive effect [43]. The nutritional recommendation for this ratio is that it should be lower than 4, and the strategy based on the replacement of animal fat by OG or EG produced a drastic decrease to values close to 1 in the PUFA n-6/n-3 ratio in the RF/OG and RF/EG fuets, complying with the recommendations. Increasing the PUFA/SFA ratio as well as reducing the PUFA n-6 / n-3 ratio to get closer to the reference values, has been tested using new lipid materials such as EG or oleogels elaborated with oils that have a healthy profile of fatty acids (olive, flax, chia, etc.). This strategy, which has been tried on other types of meat products (fermented, cooked or fresh), has given similar results to those obtained in the present study [6,8,17,18,24].

According to the composition specified, the energy value of the normal-fat fuets (NF/C) was approximately 392 kcal/100 g. As a consequence of the reformulation strategies based on lipid content improvement, the energy value decreased to values between 328 kcal/100 g (RF/OG and RF/C samples) and 338 kcal/100 g in fuet with emulsion gel (RF/EG). These changes represent an energy reduction of around 14–16% in the reformulated products. Similar or lower energy reductions have been observed in other reduced-fat fermented sausages [24,37].

### 3.3. Nutritional and Health Claims

According to the composition presented in Table 2 and Regulation (European Commission) no 1924/2006 and Regulation (EU) no 432/2012 [44,45], all the fuets could be labelled with the nutritional claim “high protein content” and the corresponding health claims presented in Table 3. On the other hand, the sample with reduced all-animal-fat content (RF/C) showed a fat reduction of more than 30% with respect to the control and could therefore labelled with a “reduced fat content” claim.

Furthermore, the strategy based on partial replacement of animal fat by healthy structured oil systems (OG and EG) allows other nutritional and health claims for these fuets according to European regulations [44,45]. With regard to nutritional claims, the RF/OG and RF/EG fuets could be labelled with “high unsaturated fat” and “high omega-3 fatty acids” claims (Table 3). With regard to health claims, the labelling of these samples could include the claim that “ALA contributes to the maintenance of normal blood cholesterol levels” (Information shall be given to the consumer that the beneficial effect is obtained with a daily intake of 2 g of ALA). Taking into account that it is recommended to limit the consumption of processed meat to 50 g per day [46], this amount of the RF/OG and RF/EG samples covers more than 50% of ALA needs. Accordingly, the presence of chia and olive oil oleogel or emulsion gel in the fuets reflected healthier nutritional properties when compared with the control samples.

### 3.4. Technological Properties

In order to know the consequences of the different composition of the fuets as well as the phenomena that occurred during the ripening process, the technological properties were evaluated during the storage period, after ripening, which is when the product is consumed. The water activity (Aw) of the fuets was affected by the formulation (Table 4), with values ranging between 0.87 and 0.90 just after the ripening period (day 0 of storage). The use of OG or EG as fat replacement in the fuets did not significantly condition the initial Aw, but their values were higher (*p* < 0.05) than those observed in the samples with all-animal fat and than those expected for this kind of product [47]. However, Triki [48] observed decreased Aw values in chorizo (a Spanish fermented sausage) fermented sausages. In the present work, what may have happened is that the use of the mixture of structured olive and chia oils in the development of the fuets conditioned the ripening process, requiring a longer time to produce a reduction in water activity levels. In general, chilled storage had hardly any effect on water activity (Table 4). Similar behavior has been observed in fermented sausages during storage [48].

The sausage formulations and chilled storage conditioned (*p* < 0.05) the pH values of the fuets (Table 4). However, all the pH values were within the normal range reported for similar commercial products [47] or products in which animal fat was replaced by n-3 long-chain PUFA in konjac glucomannan matrix or linseed EG [25,49]. At day 0, samples with OG or EG as fat replacer showed the lowest (*p* < 0.05) pH values. Similar behavior has been described for fuets in which animal fat was replaced by sunflower oil [28] and in higher caliber (50 mm) dry fermented sausages made with linseed oil EG as animal fat replacer [25]. On the other hand, [24] did not observe an effect on pH values as a consequence of fat replacement (26.3%, 32.8% and 39.5%) by linseed oil gelled emulsion in dry fermented sausages. During chilled storage a significant increase in pH values was observed. Similar results have been found in dry fermented sausage produced using different lactobacilli as starter culture [50]. These authors found that the pH started to increase after 28th day of ripening and the increase continued during storage at refrigeration (8° C). An increase of pH could be related to the breakdown of lactic acid following the depletion of the added sugar [50].

Table 4 shows the values obtained for lightness (L*), redness (a*) and yellowness (b*) in the control and reformulated fuets. As a result of reducing animal-fat content (comparison between NF/C and RF/C), increases (*p* < 0.05) in lightness and yellowness were observed, while no effect on redness values was found. However, as a result of the replacement of pork backfat by structured chia and olive oil systems (RF/OG and RF/EG samples), in comparison with the control (NF/C), only yellowness increased (*p* < 0.05) (Table 4). This means that the strategy of reducing and replacing animal fat with a mixture of structured olive and chia oils gives rise to products that maintain the characteristic redness of this type of product, unlike what happens when there is only a reduction in fat content (RF/C), which causes greater changes in colour. It is important to note that the healthier fuets (RF/OG and RF/EG samples) were more stable, with smaller changes in colour parameters after 30 days of chilled storage, than the products made with only animal fat (Table 4).

The hardness of the fuets varied as a result of the modifications that were assayed (Table 4). Initially it was noted that there was a significant increase in hardness in the fuets with reduced animal fat, probably owing to greater water losses in the RF/C samples (Figure 1). These results are in agreement with those found by several authors [28,51,52], who reported higher hardness in low-fat dry fermented sausages than in high-fat ones. The type of structured oil system used as the animal fat replacer conditioned the hardness of the fuets. Thus, fuets made with EG as animal-fat replacer (RF/EG) showed similar (*p* > 0.05) hardness to the control (NF/C), whereas those with oleogel (RF/OG) had the lowest (*p* < 0.05) hardness values. Hardness has a negative relation with moisture content in dry fermented meat products, as other authors have observed [51,53]. Accordingly, given that the RF/OG and RF/EG samples had similar moisture values (Table 2) and processing losses, the differences in hardness could be attributed to how the water was added during the preparation of the products, directly to the meat matrix (RF/OG) or stabilized in EG (RF/EG). In chorizo Jimenez-Colmenero [54] detected a decrease in hardness as a consequence of replacing various animal fat levels by an oil-in-konjac matrix. Similar behavior was observed by other authors when they used linseed oil EG or OG as an animal fat replacer in dry fermented sausages [25,26]. Conversely, in salchichón (a Spanish fermented sausage) and fuet, the replacement of various animal-fat levels by fish oil encapsulated in konjac gel (salchichón) or by sunflower oil added directly (fuet) resulted in harder samples [26,27,28]. As expected, during chilled storage all the samples experienced an increase (*p* < 0.05) in hardness (Table 4), probably because all the samples lost water during that period. However, it should be noted that, as with color, the changes in the texture of the OG and EG fuets during chilled storage were smaller than those in the control samples made with animal fat.

### 3.5. Lipid Oxidation

Lipid oxidation is the main non-microbial cause of quality deterioration in meat products and one of the most important reactions of fermented meat products that generates volatile compounds [33]. Accordingly, the effect of the partial replacement of pork backfat by structured chia and olive oil systems (oleogel or emulsion gel) on lipid oxidation, measured as volatile compounds and MDA levels, is shown in Table 5.

TBARs values were significantly higher in the RF/OG and RF/EG samples, reflecting increased lipid oxidation in the fuets owing to the higher level of unsaturated fat, although their oxidation levels remained well below the rancidity threshold which is usually when the MDA concentration is above 1 mg per kg of sample [33]. Chilled storage did not have a significant effect on TBARs values, probably because of the stability provided by the structured systems in which the oil mixture was located, unlike what occurs when the oil is incorporated directly [55]. Similar results have been found in various meat products with an improved lipid profile based on plant and marine oils stabilized in different ways [48].

Aldehydes are the most abundant volatile compounds produced by lipid oxidation, and hexanal is the aldehyde that has been considered to be the best indicator [33]. As expected, higher (*p* < 0.05) levels of all volatile compounds were observed after the ripening process (day 0) in samples with OG (RF/OG) or EG (RF/EG) used as animal fat replacer (Table 5). These results are in agreement with those obtained in the determination of TBARs and those found by some other authors. Thus, Alejandre [24] and Glisic [25] observed higher levels for aldehydes in dry fermented sausages in which the lipid content was improved by using linseed emulsion gel as an animal fat replacer. On the other hand, although RF/EG showed higher (*p* < 0.05) hexanal levels than RF/OG, non-significant differences were observed in heptanal, octanal and nonanal levels depending on the structured oil system used as healthier lipid material (Table 5). Josquin [56] assayed the replacement of pork backfat with pure, pre-emulsified or encapsulated fish oil in fermented sausages and observed differences in volatile levels, depending on the strategy used to incorporate the oil. The sausages in which encapsulated oil was incorporated had lower volatile compound levels than the others.

After chilled storage, a significant decrease was observed in the volatiles studied, except for nonanal in the samples made with OG or EG, whereas the samples with all-animal fat generally showed values (Table 5) similar to those at the beginning of storage.

### 3.6. Microbiological Analysis

**Figure 2:**
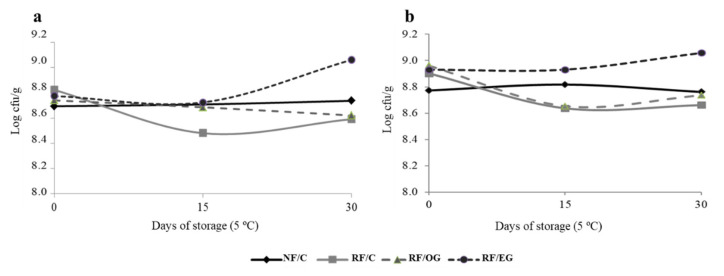
Microorganism (**a**: total viable count; **b**: lactic acid bacteria) counts (log cfu/g) of fuets during 30 days of chilled storage. For sample denominations see Table 1.

Microbiological factors during chilled storage are known to affect the stability and shelf life of meat products. Figure 2 shows changes in total viable count (TVC) and lactic acid bacteria (LAB). All samples presented high initial microbial counts (>8 log cfu/g) of TVC and LAB, which in general were maintained during chilled storage. However, fuet formulated with emulsion gel (RF/EG) experienced a significant increase in TVC and LAB counts after 30 days in refrigeration, reaching levels close to log 9 cfu/g (Figure 2). These results are in accordance with others observed in dry fermented sausages in which various animal-fat levels were replaced [35,48].

### 3.7. Sensory Analysis

**Figure 3:**
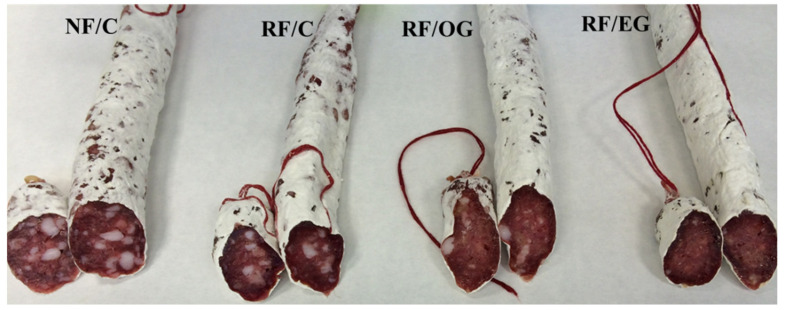
Effect of formulation strategies on the external and cross-sectional appearance of the fuets after the ripening process. For sample denominations see Table 1.

The external appearance of the fuets was similar regardless of the formulation strategy used (Figure 3). However, some differences were observed in their cross-sectional appearance, depending on the lipid source that was used. Thus, while the animal fat was perfectly differentiated in the meat matrix, the oleogel or EG in R/OG and R/EG, respectively, could not be seen (Figure 3).

**Figure 4:**
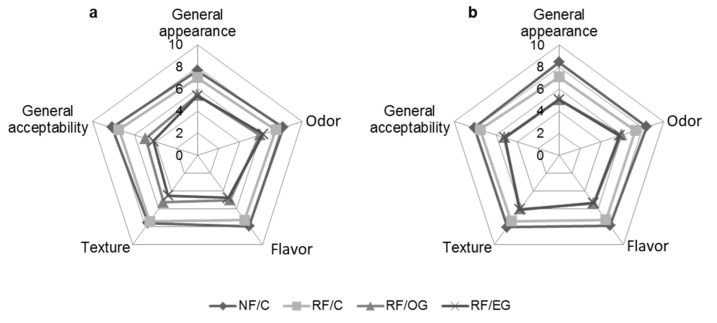
Sensory analysis scores for general appearance, odor, flavor, texture and general acceptability of the fuets: **a)** after ripening process; **b)** after 30 days of chilled storage. For sample denominations see Table 1.

The results of the hedonic analysis for the attributes evaluated are shown in Figure 4. In general, for all of them, the samples made with all-animal fat received higher scores than the others. With regard to RF/OG and RF/EG, the panelists evaluated them with similar scores for all attributes. The lower scores that the reformulated samples received could be attributed to the high aldehyde content as compared to the control (Table 5), as other authors have reported for this type of meat product [57]. On the other hand, the differences observed between their appearances (Figure 3) may have conditioned how the panelists evaluated other sensory attributes [58]. Furthermore, after 30 days of storage, when they showed lower aldehyde contents (Table 4), RF/OG and RF/EG received higher scores for flavor or general acceptability. Alejandre et al. [24] did not observe differences in taste and juiciness but found differences in odor between control dry fermented sausages and others made with linseed emulsion gel as animal-fat replacer. However, the sensory attributes could be further improved by slight modifications to the product, including modifications to the conditions associated with the ripening process.

## 4. Conclusions

The healthy oil mixture based on chia and olive oil, structured into an oleogel or emulsion gel, was proved to be an interesting option for the development of functional dry fermented sausages. These products could be labelled with certain nutritional and health claims according to European legislation, mainly because of the high α-linolenic fatty acid content. The strategy of reducing and replacing animal fat with a mixture of structured olive and chia oils gives rise to products that maintain the color characteristic of this type of product and a good oxidative and microbiological status during chilled storage. Fuets made with EG as animal-fat replacer had similar hardness to the control whereas those with oleogel were softer. Nevertheless, further studies are necessary to improve sensory attributes of the reformulated fuets with this type of lipid material but no great differences resulting from the use of one or the other were observed. Moreover, the strategy based on reduction and improvement of the lipid fraction yielded products that were stable during chilled storage.

## Figures and Tables

**Table 1:** Formulation (g/100 g) of different fuets.

	Meat	Pork Back Fat	Oleogel	Emulsion Gel	Water
NF/C	74.0	20.0			0.5
RF/C	74.0	9.0			11.5
RF/OG	74.0	4.0	7.5		9.0
RF/EG	74.0	4.0		15.0	1.5

Normal fat (NF/C) and reduced-fat (RF/C) dry fermented sausages (fuet) formulated with all-animal fat. Reduced-fat fuets reformulated by partially replacing (80%) pork backfat with oleogel (RF/OG) or emulsion gel (RF/EG). All samples contain 5.5% of special commercial seasoning preparation for fuet.

**Table 2:** Chemical compositions and nutritional significance ratios of different fuets after the ripening process.

Parameters	Samples			
	NF/C	RF/C	RF/OG	RF/EG
Composition (%)				
Moisture	32.63 ± 0.87 ^a^	36.07 ± 0.47 ^b^	37.45 ± 0.70 ^b^	36.84 ± 0.76 ^b^
Ash	4.72 ± 0.04 ^a^	6.13 ± 0.03 ^c^	4.90 ± 0.01 ^ab^	5.07 ± 0.15 ^b^
Protein	31.17 ± 0.07 ^a^	37.74 ± 0.74 ^c^	31.92 ± 0.96 ^a^	35.12 ± 0.24 ^b^
Fat	29.73 ± 2.42 ^b^	19.78 ± 1.86 ^a^	22.30 ± 2.13 ^a^	22.01 ± 0.61 ^a^
Fatty acid profile (g/100 g of fuet)				
Myristic C14:0	0.50 ± 0.01 ^c^	0.37 ± 0.04 ^b^	0.17 ± 0.00 ^a^	0.16 ± 0.01 ^a^
Palmitic C16:0	7.85 ± 0.24 ^c^	5.82 ± 0.57 ^b^	3.92 ± 0.06 ^a^	3.76 ± 0.12 ^a^
Stearic C18:0	3.54 ± 0.17 ^c^	2.63 ± 0.28 ^b^	1.56 ± 0.03 ^a^	1.55 ± 0.06 ^a^
∑ SFA	12.11 ± 0.42 ^c^	8.99 ± 0.90 ^b^	5.79 ± 0.09 ^a^	5.61 ± 0.19 ^a^
Vaccenic C18:1n7	1.19 ± 0.03 ^c^	0.91 ± 0.08 ^b^	0.63 ± 0.01 ^a^	0.62 ± 0.02 ^a^
Oleic C18:1n9	13.60 ± 0.33 ^c^	10.30 ± 0.93 ^a^	12.29 ± 0.20 ^b^	11.94 ± 0.18 ^b^
∑ MUFA	16.12 ± 0.38 ^c^	12.20 ± 1.10 ^a^	13.51 ± 0.22 ^b^	13.10 ± 0.21 ^b^
Linoleic C18:2n6	2.31 ± 0.04 ^b^	1.81 ± 0.14 ^a^	1.81 ± 0.02 ^c^	1.77 ± 0.03 ^c^
Linolenic C18:3n3	0.12 ± 0.00 ^a^	0.09 ± 0.01 ^a^	1.68 ± 0.03 ^b^	1.61 ± 0.03 ^b^
∑ PUFA	2.83 ± 0.05 ^b^	2.25 ± 0.17 ^a^	3.81 ± 0.04 ^d^	3.60 ± 0.04 ^c^
Nutritional significance ratios				
PUFA/SFA	0.23 ± 0.01 ^a^	0.25 ± 0.01 ^a^	0.66 ± 0.01 ^b^	0.64 ± 0.02 ^b^
n-6/n-3	14.0 ± 0.22 ^b^	15.04 ± 0.47 ^b^	1.12 ± 0.01 ^a^	1.19 ± 0.02 ^a^

SFA: saturated fatty acid; MUFA: monounsaturated fatty acid; PUFA: polyunsaturated fatty acid. For sample denominations, see Table 1. Different letters in the same row indicate significant differences (*p* < 0.05) between formulations. Means ± standard deviation.

**Table 3:** Nutrition and health claims authorised in fuets according to Regulation (EC) No 1924/2006 and Commission Regulation No 432/2012.

Claims	Conditions Applying to Them	Fuet Samples			
		NF/C	RF/C	RF/OG	RF/EG
“high protein” Proteins contribute to a growth in muscle mass and the maintenance of muscle mass and normal bones. Protein is needed for normal growth and development of bone in children.	May only be made where at least 20% of the energy value of the food is provided by protein	X	X	X	X
“reduced fat”	May only be made where the reduction in content is at least 30% compared to a similar product		X		
“high unsaturated fat” Replacing saturated fats with unsaturated fats in the diet contributes to the maintenance of normal blood cholesterol levels.	May only be made where at least 70% of fatty acids present in the product derive from unsaturated fat under the condition that unsaturated fat provides more than 20% of the energy of the product.			X	X
“high omega-3 fatty acids” Alpha-linolenic acid (ALA) contributes to the maintenance of normal blood cholesterol levels. Information shall be given to the consumer that the beneficial effect is obtained with a daily intake of 2 g of ALA.	May only be made where the product contains at least 0.6 g ALA / 100 g of product and per 100 kcal.			X	X

**Table 4:** Technological properties of fuets during chilled storage: pH and water activity (Aw) values, colour parameters (L* lightness, a* redness and b* yellowness) and texture profile analysis (TPA) (Hardness, N).

	*Samples*	*Days of Storage (5 °C)*		
		*0*	*15*	*30*
*Aw*	NF/C	0.87 ± 0.02 ^a1^	0.89 ± 0.01 ^c1^	0.88 ± 0.00 ^a1^
	RF/C	0.88 ± 0.01 ^a2^	0.84 ± 0.00 ^a1^	0.87 ± 0.00 ^a2^
	RF/OG	0.91 ± 0.01 ^b1^	0.90 ± 0.00 ^c1^	0.90 ± 0.00 ^b1^
	RF/EG	0.90 ± 0.01 ^b2^	0.88 ± 0.01 ^b1^	0.92 ± 0.00 ^b3^
*pH*	NF/C	5.41 ± 0.01 ^b1^	5.74 ± 0.11 ^b2^	6.34 ± 0.04 ^a3^
	RF/C	5.50 ± 0.01 ^b1^	5.87 ± 0.07 ^c2^	6.5 ± 0.04 ^b3^
	RF/OG	5.27 ± 0.01 ^a1^	5.60 ± 0.04 ^a2^	6.64 ± 0.03 ^c3^
	RF/EG	5.20 ± 0.01 ^a1^	5.77 ± 0.12 ^bc2^	6.62 ± 0.02 ^c3^
*Colour parameters*				
L*	NF/C	41.45 ± 2.85 ^a12^	42.74 ± 2.99 ^a2^	38.76 ± 1.59^ab1^
	RF/C	45.68 ± 2.79 ^b2^	41.02 ± 1.63 ^a1^	39.60 ± 3.99 ^b1^
	RF/OG	41.69 ± 2.47 ^a1^	41.69 ± 1.92 ^a1^	40.76 ± 2.39 ^b1^
	RF/EG	42.12 ± 1.86 ^ab2^	39.85 ± 2.52 ^a2^	35.31 ± 3.50 ^a1^
a*	NF/C	14.11 ± 0.99 ^ab2^	11.60 ± 2.99 ^a1^	17.75 ± 1.73 ^b3^
	RF/C	12.79 ± 1.87 ^a1^	16.82 ± 1.17 ^b2^	16.72 ± 1.70 ^ab2^
	RF/OG	15.58 ± 1.38 ^b1^	16.75 ± 0.88 ^b1^	16.74 ± 2.04 ^ab1^
	RF/EG	14.25 ± 1.29 ^ab1^	16.52 ± 1.01 ^b2^	15.33 ± 0.95 ^a12^
b*	NF/C	6.07 ± 0.82 ^a1^	4.66 ± 1.39 ^a1^	7.77 ± 1.36 ^a2^
	RF/C	8.23 ± 2.40 ^b1^	7.01 ± 1.19 ^b1^	7.66 ± 0.83 ^a1^
	RF/OG	7.91 ± 0.98 ^b1^	10.19 ± 1.08 ^c2^	10.43 ± 1.10 ^b2^
	RF/EG	8.95 ± 0.78 ^b1^	9.70 ± 0.74 ^c1^	9.32 ± 0.82 ^ab1^
*Texture profile analysis*				
*Hardness (N)*	NF/C	5.81 ± 0.91 ^b1^	4.54 ± 1.40 ^a1^	12.30 ± 1.66 ^b2^
	RF/C	9.51 ± 0.18 ^c1^	12.36 ± 2.06 ^c1^	16.79 ± 3.34 ^c2^
	RF/OG	3.75 ± 0.49 ^a1^	3.78 ± 0.65 ^a1^	5.36 ± 0.52 ^a2^
	RF/EG	5.84 ± 0.50 ^b1^	7.71 ± 1.08 ^b2^	7.97 ± 0.73 ^a2^

For sample denominations, see Table 1. Different letters in the same column and different number in the same row indicate significant differences (*p* < 0.05) between formulations or chilled storage process. Means ± standard deviation.

**Table 5:** Parameters related to lipid oxidation of fuets during chilled storage: thiobarbituric acid-reactive substances (TBARs) values (mg malonaldehyde (MDA)/kg sample) and volatile compounds (area/sample weight (g) × 10^3^).

*Compound*	*Samples*			
	*NF/C*	*RF/C*	*RF/OG*	*RF/EG*
*TBARs*				
*day 0*	0.103 ± 0.025 ^a1^	0.085 ± 0.010 ^a1^	0.404 ± 0.028 ^b1^	0.404 ± 0.028 ^b1^
*day 30*	0.107 ± 0.016 ^a1^	0.091 ± 0.010 ^a1^	0.384 ± 0.040 ^b1^	0.365 ± 0.041^b1^
*Hexanal*				
*day 0*	156.0 ± 0.4 ^a1^	152.3 ± 20.8 ^a1^	239.8 ± 26.2 ^b2^	367.0 ± 15.2 ^c2^
*day 30*	119.4 ± 18.4 ^a1^	121.3 ± 9.9 ^a1^	153.1 ± 0.5 ^a1^	279.4 ± 26.2 ^b1^
*Heptanal*				
*day 0*	17.5 ± 3.1 ^a2^	30.8 ± 4.8 ^a2^	65.3 ± 36.8 ^b2^	60.1 ± 3.8 ^b2^
*day 30*	11.9 ± 1.3 ^a1^	12.3 ± 5.6 ^a1^	35.6 ± 1.1 ^b1^	48.0 ± 1.2 ^c1^
*Octanal*				
*day 0*	74.2 ± 4.0 ^a1^	55.4 ± 1.9 ^a1^	473.3 ± 188.5 ^b2^	462.2 ± 139.3 ^b2^
*day 30*	51.4 ± 3.4 ^a1^	51.0 ± 6.1 ^a1^	96.5 ± 0.5 ^b1^	137.9 ± 11.5 ^c1^
*Nonanal*				
*day 0*	366.9 ± 20.7 ^a1^	352.6 ± 39.4 ^a1^	971.8 ± 164.4 ^b1^	809.2 ± 1.1 ^b1^
*day 30*	324.9 ± 18.1 ^a1^	387.8 ± 66.2 ^a1^	762.2 ± 22.3 ^b1^	882.5 ± 20.9 ^b1^

For sample denominations, see Table 1. Different letters in the same row indicate significant differences by formulation and different number in the same column indicate differences by chilled storage (*p* < 0.05). Means ± standard deviation.
